# Telerehabilitation in Colombia: A Country Report and Qualitative Analysis During the Covid-19 Pandemic - Perceptions of Healthcare Providers and Patients

**DOI:** 10.5195/ijt.2024.6605

**Published:** 2024-06-28

**Authors:** Sara Gabriela Pacichana-Quinayaz, Lina María Rodríguez Vélez, Daniel Sánchez Cano, Yisel Mabirlly León Sánchez, Olga Marina Hernández Orobio, Maria Ana Tovar Sánchez, Gloria Isabel Toro Córdoba, Francisco Javier Bonilla-Escobar

**Affiliations:** 1 Research Group in Physical Medicine and Rehabilitation at Universidad Del Valle (GIRUV), Department of Physical Medicine and Rehabilitation, Cali, Colombia; 2 Research Group Curriculum and Pedagogy in Higher Education (CURPES), Universidad Del Valle, Cali, Colombia; 3 Fundación Somos Ciencia al Servicio de la Comunidad, Fundación SCISCO/Science to Serve the Community, SCISCO Foundation, Cali, Colombia; 4 Institute for Clinical Research Education, School of Medicine, University of Pittsburgh, Pittsburgh, USA

**Keywords:** COVID-19, Qualitative research, Telehealth, Telemedicine, Telerehabilitation

## Abstract

Given Colombia's status as a middle-income country with healthcare challenges, leveraging telemedicine could significantly benefit hard-to-reach regions, under-resourced and underserved communities. This study provides a comprehensive overview of the country's telerehabilitation landscape, exploring the clinicians' and patients' perspectives during the COVID-19 pandemic through systematic review and qualitative analysis. Sixteen therapists and three patients were identified via snowball sampling. The literature review was scarce and scattered across various topics in the country; some studies delved into specific aspects like legislative frameworks and patient outcomes from different medical specialties. The qualitative analysis demonstrates that despite the learning curve, telerehabilitation strengthens therapeutic support, enhances patient autonomy, fosters a positive patient-provider relationship, achieves treatment goals, promotes family involvement, reduces time and costs, and ensures continuity of therapy services. This study identified research gaps, challenges, and opportunities in telerehabilitation in a Latin American country. Adopting telemedicine technologies in low- and middle-income countries could significantly enhance their healthcare systems.

## About Colombia

Colombia, positioned in the northern region of South America, is a sizable country spanning approximately 1.14 million square kilometers, with an estimated population of 51 million people. Colombia is among the low- to middle-income countries in South America with a growing economy (CEDLAS & The World Bank, 2021). The demographic landscape is characterized by a distribution between rural and metropolitan areas, with a noteworthy proportion residing in urban areas (57%) and rural regions covering most of the municipalities in the country (89%) (Organisation for Economic Co-operation and Development, 2022).

The nation's social security health system is structured around subsidiary and contributorship subsystems. Notably, subsidiaries constitute a significant percentage, accounting for approximately 55% percent. Concurrently, 13% of the population is situated below the poverty datum line, highlighting prevalent economic challenges experienced by a substantial segment (DANE, 2023; Mora-Moreo et al., 2023).

A conspicuous concern lies in the maldistribution of wealth, where a minority accumulates most of the country's resources and 0.4% of the population owns 62% of the country's best land. This socioeconomic imbalance poses challenges, hindering technology and resources from effectively reaching the more geographically distant and underserved segments of the population (Gillin, 2015).

Considering these circumstances, our intent was to construct a qualitative country report, focusing on a telerehabilitation project implemented during the COVID-19 pandemic.

## Telehealth and COVID-19 in Colombia

The COVID-19 pandemic ushered in disruptions in healthcare service delivery marked by mandatory isolation measures, the prioritization of vulnerable populations, and restrictions in accessing essential medical services (Trejos-Herrera et al., 2020; World Health Organization, 2020). The continuity of rehabilitation services (Ambrosino et al., 2021) through the use of healthcare technologies such as telehealth emerged as an imperative component to navigate these challenging circumstances (Gallegos, 2020).

Pre-pandemic, Colombia was already grappling with a crisis in rehabilitation care, particularly in the context of minority populations, displaced individuals, victims of armed conflict, and those confronted with limited access to education and income (Álvarez Castaño, 2009; Eraso, 2015; Gómez Acosta & Cuervo Echeverri, 2007; Ospina & Serrano, 2010). The onset of the pandemic further exacerbated these existing issues, intensifying challenges surrounding the implementation of telehealth due to regulatory gaps, infrastructure limitations, a shortage of human resources, and sustainability concerns (Boldrini et al., 2020; Boyle et al., 2020; de Sire et al., 2020).

Among those severely impacted by these developments were individuals with disabilities or those necessitating physical therapy and/or other rehabilitation services (Ambrosino et al., 2021; de Sire et al., 2020). They were already experiencing health and socioeconomic disparities (Boyle et al., 2020), therefore, making them even more vulnerable to the ravages of the pandemic (Leocani et al., 2020). Factors like the fear of contagion, the absence of specialized care, and difficulties in accessing services, including healthcare, gave rise to gaps and impediments in disability care and rehabilitation (Boldrini et al., 2020; Boyle et al., 2020; Jalali et al., 2020).

Comprehending the challenges of telerehabilitation from the vantage points of patients and healthcare professionals is imperative (Sugarman et al., 2021). Such insights can offer critical assistance in advancing telehealth implementation and enhancing the quality of services. A qualitative approach to understanding telerehabilitation programs can yield invaluable information for tailoring more effective and gratifying healthcare services (Marín Urrego, 2023; Qvarfordt et al., 2023). The experiences and expertise of providers offer valuable insights into optimizing telerehabilitation sessions, personalizing interventions, and addressing challenges (de Varge Maldonado et al., 2023). Furthermore, involving providers in the planning and design of programs fosters a collaborative environment that fuels innovation and growth (Narváez et al., 2017).

To the best of our knowledge, no studies have explored the context of telerehabilitation in the country, as well as the perceptions of patients or healthcare service providers regarding the telerehabilitation process during a pandemic (ClinicalTrials.gov; Marks et al., 2022). In Colombia, telerehabilitation is still an emerging service delivery model that has facilitated access to rehabilitation services for a population characterized by conditions of vulnerability and/or disabilities. Comprehending this process presents an opportunity for innovation and the optimization of rehabilitation services disrupted during the pandemic (Gallegos, 2020).

This study aimed to characterize the telerehabilitation services in the country while considering implications during a pandemic, from the perspectives of patients, caregivers, and healthcare providers in healthcare institutions. The primary purpose was to report the country's telerehabilitation scenario and identify patients and caregiver perceptions regarding this service delivery model through qualitative data analysis, while addressing the challenges in accessibility to rehabilitation services. These findings have the potential to be utilized for the improvement of telerehabilitation services and outreach in future settings and comparable economies.

## Methodology

### Study Type

This is an exploratory country report through a literature review and a qualitative study conducted through in-depth interviews with rehabilitation practitioners in Cali, Colombia. It delves into their experiences and engagement in telerehabilitation services, thoroughly investigating the main challenges encountered during the obligatory lockdown period prompted by the COVID-19 pandemic.

### Study Area

This research was carried out in a prominent city in the southwestern region of Colombia renowned for its provision of telerehabilitation services. Local health authorities have actively pursued a deeper understanding of telerehabilitation in recent times (Camacho, 2020).

### Population and Sample

Nineteen participants were recruited, in adherence to the fundamental tenets of category saturation and information representation (Scott & Mars, 2020). These participants were consciously sourced from reference telerehabilitation centers situated in the southwestern region (Fundacion IDEAL, 2022) using a snowball sampling technique. This enabled a progressive selection of participants as key individuals. They were identified and references obtained based on their experience in conducting telerehabilitation interventions (i.e., healthcare professionals) or as subjects of intervention (i.e., patients and caregivers) during the pandemic. In some cases, professionals were also referenced because they had training or certification in telerehabilitation. The participants assumed roles as service providers such as speech-language pathologists, physical therapists, physiatrists, and occupational therapists, alongside patients who were actively engaged in the telerehabilitation process. Within this cohort, 16 individuals were healthcare professionals, and three were patients.

## Data Collection

### Literature Review

The abstracts of all located papers were subjected to review by two independent assessors. We included papers featuring clinical data, epidemiological studies (evaluating or describing telerehabilitation services in any form such as physiotherapy, occupational therapy, speech-language therapy, or others) during the pandemic. Furthermore, we included studies reporting outcomes related to effectiveness, safety, patient satisfaction, or other relevant telerehabilitation outcomes in Colombia.

Excluded were papers published in languages other than Spanish or English, unless translation was provided. Studies in the early stages of technological development without applicable clinical outcomes were also excluded. Disputes regarding the suitability of a paper were resolved through discussion, and if consensus was not achieved, the paper was excluded.

**Figure 1:**
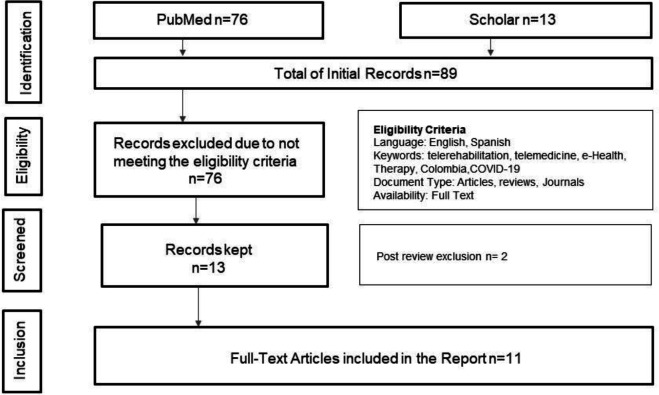
Systematic Process Used in the Selection of Articles, Based on PRISMA (Preferred Reporting Items for Systematic Reviews and Meta-Analyses)

We searched the following electronic databases: PubMed, PMC, and Google Scholar. We searched for papers published from June 15, 2018-January 15, 2024. The process is shown in Figure 1.

The search strategy included terms as: “Physical Therapy Modalities” OR “Physical Therapy (Specialty)” OR “Physical Therapy Department, Hospital” OR “Occupational Therapy” OR “Occupational Therapy Department, Hospital” OR “Speech Therapy” AND “Telemedicine” OR “Telecommunications” OR “Remote Consultation” OR “Computer Communication Networks” OR “Electronic Mail” OR “Videoconferencing” OR “Telerehabilitation” OR “Telepractice” OR “Teleorientation” OR “COVID-19” OR “Colombia.”

This comprehensive approach was adopted to contextualize the landscape of telehealth services in the country. We aimed to compare and enrich these services for the future, leveraging insights gained from our concurrent qualitative approach.

### Qualitative Approach

An interview guide structured around four primary inquiry axes was employed to delve into key areas encompassing: the experience and adaptation to telerehabilitation, the logistical and regulatory facets of implementation, the primary challenges encountered during implementation, and the diverse experiences and perspectives of different stakeholders.

The interviews were conducted via both traditional in-person methods and modern virtual platforms, including Zoom and Google Meet. After securing informed consent from each participant, comprehensive interviews were conducted, with an average duration of approximately 30 minutes. The interviews were conducted using clear and simple language to ensure effective communication with the respondents.

Given the qualitative nature of this study, the determination of the number of interviews included was guided by data saturation, which signifies the point at which no novel information was forthcoming from the participants (Guest et al., 2006). The interviews were recorded in MP3 audio format, transcribed using Microsoft Word, and subjected to analysis through the utilization of the qualitative software AtlasTi version 9 ®.

### Analysis

A thematic analysis of the pre-established categories was undertaken, elucidating the nuanced interpretations provided by the interviewees concerning their lived experiences and operational procedures during the telerehabilitation implementation, with due consideration of their distinct roles. This analysis involved the initial coding of the categories, a procedure that discerned the fundamental ideas and concepts about the telerehabilitation process, which was embedded within the interviews.

Furthermore, a thematic coding was carried out. This illuminated the intricate connections, patterns, resemblances, and disparities that emerged among the identified conceptual elements, and facilitated a nuanced exploration of the perspectives presented by the participants. Researchers carried out triangulation by independently analyzing the information and then cross-verifying their individual analysis, offering a more comprehensive and robust understanding of the diverse intricacies within the realm of telerehabilitation experiences and practices.

### Ethical Considerations

This study obtained ethical approval from the institutional review board of the participating institutions.

## Results

### Literature Review

The initial search yielded 89 papers, and the search process is illustrated in Figure 1. Following the assessment, 11 papers were included as they fulfilled the inclusion criteria. The selected papers covered various topics. While some discussed the implementation of telemedicine or telerehabilitation, the incorporation of experience in Colombia was rare.

The available information on telerehabilitation in Colombia appears to be limited and dispersed across various topics, encompassing their implementation in different settings, with encountered barriers, and potential implications for the future. One study conducted a systematic review of telerehabilitation services in rural communities in Colombia (Castillo et al., 2023). Additionally, two studies provided narrative reviews from distinct perspectives during COVID-19 (Figueroa, 2020; Michell et al., 2022), while two others delved into the legislative aspects surrounding telemedicine in the country, outlining its requirements and limitations (García-Pereáñez & García-Arango, 2021; Pereáñez et al., 2019).

Moreover, four articles presented original research, exploring the potential implications of telemedicine in patients with chronic diseases such as lupus, rheumatoid arthritis (RA), and maternal and prenatal outcomes (Aryananda et al., 2023; Escobar et al., 2023; Hernández-Zambrano et al., 2021; Savedoff et al., 2023). Another study proposed a telerehabilitation engineering model for synchronized attention in rural areas (Rubiano-Ovalle et al., 2022). Lastly, a reflexive study addressed the imperative need to enhance speech-language and occupational therapy through healthcare policies and new technologies (Moreno-Chaparro et al., 2018).

We identified in these studies some aspects requiring further discussion, such as the diverse focus of the studies, the potential and challenges of telerehabilitation, and the holistic approach to healthcare enhancement.

### Qualitative Study

The average age of the professional group was 34±6 years. Among them, 69% (11) were female, and 31% were male (5). The majority were speech-language pathologists, comprising 44% of the group (7 professionals), followed by physical therapists at 31% (5 professionals), and occupational therapists at 19% (3 professionals). Additionally, there was one physiatrist who served as a virtual consultation physician.

Four distinct analysis categories were meticulously examined during this study, each offering unique insights into the realm of telerehabilitation:

**Adaptability to Telerehabilitation:** This category delves into the participants' capacity to adapt to the telerehabilitation framework.

**Continuity of Rehabilitation:** Focusing on the seamless continuation of rehabilitation processes through telehealth.

**Challenges in Telerehabilitation Development:** Identifying and addressing the various challenges encountered in the development and implementation of telerehabilitation services.

**Collaborative Work:** This category examines the collaborative efforts and dynamics within the telerehabilitation context, emphasizing teamwork and mutual contributions.

Within these primary analysis categories, numerous subcategories were employed to further refine the classification and analysis of the gathered information, as illustrated in Figure 1. The insights derived from the interviews were instrumental in the construction and elucidation of these analytical categories.

**Figure 2:**
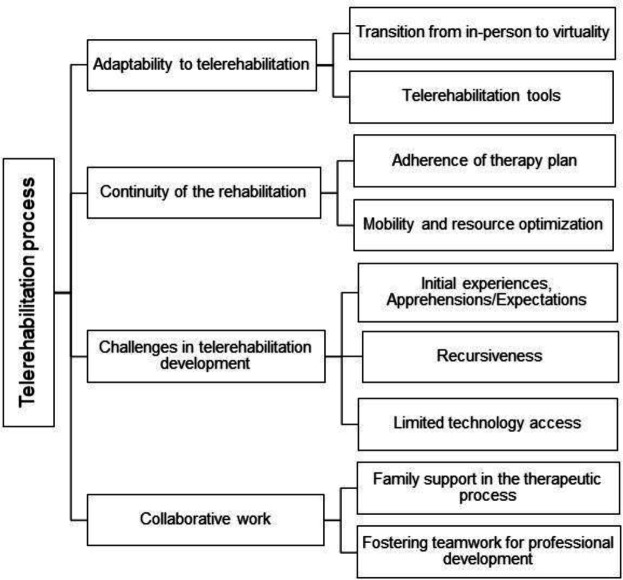
Analysis Categories

### Adaptability to Telerehabilitation Processes

#### Transition from In-person to Virtuality

Professionals stressed the need for effective exercise monitoring to ensure rehabilitation continuity. Our findings revealed varying readiness levels among institutions and professionals when it came to adapting to telerehabilitation. For instance, one institution had a range of digital tools already in place due to prior experience with remote programs, utilizing their platform for video call interventions. Conversely, several institutions and independent professionals had a more gradual integration of telerehabilitation. While it's commonly believed that “technology is accessible to everyone,” many individuals faced challenges related to limited internet access, device availability, and technological device usage.

*“…the telerehabilitation and telemedicine model had already been implemented for remote areas (in the clinic), so it wasn't a new project; it was being carried out for consultations with specialist doctors in remote regions…”* (Professional 1, 2022)

However, some institutions lacked the necessary digital infrastructure for telerehabilitation. This led to collaborative efforts between institutions and healthcare professionals to devise and implement innovative intervention strategies, adapting to the global health crisis while ensuring ongoing patient care. Initially, some healthcare providers had reservations about virtual solutions due to platform limitations, but their perspectives evolved over a few months, leading them to consider the practicality of this approach.

*“The transition was quite challenging because we went from working in a spacious area with equipment to in-person patients, sitting in with a computer and attempting to provide neurorehabilitation to patients.”* (Professional 2, 2022)

#### Telerehabilitation Tools

In the realm of telerehabilitation, professionals necessitated specific technological and methodological tools to deliver care and therapy effectively, in addition to formulating home exercise regimens for users. In this context, essential technological requisites for both the professional and the patient included internet connectivity, a camera-equipped device for video calls, an appropriate space for therapy sessions, and adequate lighting, among other considerations.

*“…at first, I didn't know how to use the platforms, Although I received training, I learned other things independently. So, I had to figure out which activities were most useful, and some strategies such as making my voice deeper for patients to understand me.”* (Professional 3, 2022)

Conversely, institutions initiated the development of virtual learning strategies, primarily due to the limited prior experience of some professionals and patients with virtual platforms. Additionally, there was a pronounced emphasis on fostering effective communication between the professional and the patient, alongside the provision of clear and concise instructions for exercises. Moreover, professionals leveraged visual and audiovisual aids, including interactive games and instructional videos to enhance the engagement and effectiveness of telerehabilitation in the patient's recovery.

*“… About the instructions we received through the clinic platform, sometimes it failed, however, it was good to implement the use of images and visuals to guide us, and we already know how to perform certain movements and exercises, given that they were like those performed in person…”* (Patient 1, 2022)

### Continuity of Rehabilitation Process

#### Adherence to the Therapy Plan

Professionals emphasized the significance of maintaining continuity in patients' treatment plans throughout the pandemic. To ensure this, they conducted virtual follow-up sessions and therapies. In some instances, due to connectivity limitations, these follow-ups were conducted via telephone for patients who could not support video calls effectively.

*“Achieving the therapeutic objectives depended on the continuity of treatment, this was independent of whether it was teleconsultation or in person, the important thing was that the patient resumed therapy. those who did it achieved their goals.”* (Professional 1, 2022)

#### Mobility and Resource Optimization

In patient interviews, participants conveyed that transportation posed challenges, highlighting that telerehabilitation enabled them to realize cost savings without disrupting the continuity of their treatment. Professionals similarly acknowledged that these virtual consultations extended coverage to remote or hard-to-reach regions. Users also noted that the elimination of travel time optimized the efficiency of their consultations and in-home treatments.

*“…transportation, that was very traumatic in my case before telerehabilitation, my wife had to ask for permission at work every day to take me to the therapies…”* (Patient 2, 2022)

*“For patients who have mobility difficulties, bedridden, elderly, or those who couldn't get vaccinated, it was very difficult for them to go out. For them, telerehabilitation makes things much easier”* (Professional 4, 2022)

*“I saved time, I needed assistance to be lifted, to get into a wheelchair, and then to find a parking space. So, those kinds of things, I think, were beneficial”* (Patient 3, 2022)

### Challenges in Telerehabilitation Development

#### Initial Experiences, Apprehensions, and Expectations

In the early stages, professionals had concerns about effectively conducting remote therapy and providing instructions to patients' caregivers or family members. Their main worries centered around potential injuries or incorrect exercise performance. However, over time, they reported significant learning, increased patient engagement, stronger commitment from family members and caregivers, and notable therapeutic improvements. The initial concerns surrounding telerehabilitation gradually eased with experience, reinforcing activities conducted outside the consultation setting and within patients' homes.

*“Something I've tried to do since I had the tele orientation experience is to switch roles. That the caregiver not only listens to the instruction, but also practices it with the patient, while I guide and supervise the therapy.”* (Professional 7, 2022)

*“We observed progress with a patient in a wheelchair. Through telerehabilitation, we worked on functional transfers, and the patient continued these exercises at home. Upon returning for an in-person session, they had made significant strides, achieving independent walking."* (Professional 9, 2022)

#### Recursiveness

Both professionals and patients encountered specific challenges and difficulties. Therapists noted the difficulty of engaging patients' attention, particularly in cases with neurological involvement, and ensuring effective communication. Many professionals strived to establish clear and efficient visual and auditory communication to deliver instructions effectively. They also needed to adjust interventions to make use of the resources available within patients' homes, which included technology, furniture, and various items employed during therapy sessions.

*“It was about the instruction, understanding how to transmit it, what type of words or images to use so that the patient and/or their caregiver could execute it correctly.”* (Professional 6, 2022)

*“Some diagnoses, such as autism, pose a challenge in telemedicine. Although we've had some success with them, it requires a lot of family support”* (Professional 7, 2022)

Similarly, users highlighted the challenge of comprehending instructions through a virtual medium. Nevertheless, they acknowledged that this process empowered them with greater autonomy because, even though professionals supplied instructions, their progress and therapy hinged on their own consistency and practice at home.

#### Limited Technology Access

Limitations in access to technology, communication devices, internet connectivity, among others, was another challenging item, as some patients occasionally experienced communication issues or interruptions during therapy sessions, or they lacked devices or computers that support platforms and all elements of virtual interaction. In some cases, professionals had to resort to alternatives such as making videos with instructions, and patients also had to record the exercises performed.

*“We had Internet issues, unstable but not entirely offline, which hindered teleconsultations. To cope, the therapist had to record videos, send them to me, and I'd reciprocate by recording myself doing the exercises. Frequent interruptions and video pause due to poor Internet made it challenging.”* (Patient 2, 2022)

### Collaborative Work

#### Family Support in Therapeutic Process

Throughout, telerehabilitation reinforced family involvement in therapy, offering professionals insights into patients' circumstances, conditions, and quality of life. This enhanced teamwork and reshaped professionals' perceptions, highlighting telerehabilitation's effectiveness with family and patient support. However, professionals encountered patients and caregivers who did not fully engage, impacting patient progress. While goals were achieved, improvement was slower than in-person therapy, partly due to the lack of initial physical assessment.

*“I observed that parents became more engaged and gained a better understanding of their child's condition during the telerehabilitation process. They learned how to effectively manage it, which is beneficial as the family's involvement significantly contributes to the patient's progress” (*Professional 8, 2022).

#### Fostering Teamwork for Professional Development

During the contingency, professionals received institutional support to oversee their processes and promote collaboration among institutions, professionals, and patients. This support included providing a telerehabilitation consultation platform and conducting training sessions to enhance professionals' abilities to comply with regulations.

However, in some institutions, the transition to this service mode was in its early stages due to their unpreparedness for the contingency, necessitating significant organizational and administrative efforts to ensure quality patient care.

*“There was prior preparation, with organized cell phones and everything ready, enabling interaction and support network integration. Caregivers actively assisted dependent individuals during therapy, fostering motivation throughout these experiences."* (Professional 1, 2022)

## Discussion

The COVID-19 pandemic and the subsequent lockdown reshaped healthcare locally and globally. Strategies to improve healthcare access and timeliness by leveraging technology, especially in rehabilitation and virtual therapy, played a crucial role, giving rise to telerehabilitation services (Scott & Mars, 2020).

The existing literature on telemedicine and telerehabilitation in Colombia is limited and exhibits a diverse range of perspectives. Numerous studies adopt unique approaches to telemedicine, encompassing areas such as comparative assessments of telerehabilitation services in different settings (gynecology, pediatrics, speech and occupational therapy) and considerations of regulatory challenges amid the COVID-19 pandemic (Aryananda et al., 2023; Castillo et al., 2023; Escobar et al., 2023; Figueroa, 2020; Moreno-Chaparro et al., 2018). Nevertheless, none of the reviewed articles delved into a qualitative perspective involving physicians, caregivers, and patients receiving telerehabilitation services. We believe our work in this area could provide valuable insights and positively contribute to the continuously evolving field.

A noteworthy systematic review conducted in 2023 underscores the profound influence of telemedicine and e-Health applications within the Colombian healthcare landscape, with a particular emphasis on their transformative role in rural communities. This comprehensive toolset proves instrumental in amplifying the identification and management of chronic conditions, such as cognitive decline and cardiovascular issues (Castillo et al., 2023). However, multiple challenges emerged as statistics indicate that despite the increased usage of telemedicine after COVID-19, it was less utilized in the subsidized subsystem, possibly due to a lack of technological resources within this population. This underscores the pressing need to address this disparity, as individuals within the subsidized population stand to benefit significantly from this model of healthcare delivery (Rubiano-Ovalle et al., 2022; Savedoff et al., 2023).

Moreover, the regulatory legislation on telemedicine is in its early stages, requiring reforms to align with the needs of the population. This is essential not only in response to the challenges posed by COVID-19 but also to extend the reach of telemedicine and telerehabilitation to populations unable to relocate, such as inmates (García-Pereáñez & García-Arango, 2021; Pereáñez et al., 2019). A significant reflection from Moreno-Chaparro et al, on healthcare policies, particularly the “Zero to Forever” initiative, which focuses on the optimal development of children from the perspective of speech-language pathology and occupational therapy, explored various avenues to enhance children's health through political measures, guidelines, and innovative technologies such as telemedicine (Moreno-Chaparro et al., 2018).

In the comparative study by Michell et al. (2022), the researchers delved into telerehabilitation services for physiotherapy across South America (including Colombia) and Australia. It revealed heightened barriers in underdeveloped countries in terms of resources, infrastructure, technologies, and networks required for adequate development. However, it concurrently emphasized substantial opportunities for healthcare advancements, as telerehabilitation showed promise for healthcare (Michell et al., 2022). However, as highlighted earlier, the literature in Colombia is limited, as most of these studies were conducted in high-income countries, emphasizing the lack of research in middle and low-income countries (Begazo Flores et al., 2023; Marks et al., 2022). The adaptation to new technologies and virtual platforms by healthcare personnel takes time to ensure the proper development of therapy sessions (Coronel et al., 2022).

While not all institutions were initially prepared for virtual care, pre-existing telehealth programs played a pivotal role in adapting to telerehabilitation. They extended care coverage and facilitated substantial therapeutic development through collaborative networking (Silva-Tinoco Rubén, 2021).

Providing remote care through virtual platforms significantly enhanced rehabilitation during the pandemic, ensuring equitable access to services, particularly for those in remote areas or with difficulties accessing in-person healthcare facilities (Chang et al., 2021; Coronel et al., 2022; Silva-Tinoco Rubén, 2021). However, the absence of an initial in-person clinical assessment can hinder therapeutic goal establishment, particularly for older adults or patients with disabilities, necessitating additional support for participation in virtual interventions (Haleem et al., 2021).

The integration of telehealth during the pandemic demanded a steep learning curve, with healthcare professionals and patients continually adapting to virtual tools to ensure the provision and receipt of remote care, aligning with social distancing guidelines (Avendaño Veloso et al., 2022; Gobierno de Colombia, 2019). Professionals reported improving development and achieving therapy goals with each telemedicine/rehabilitation session, highlighting the rapid learning curve in telehealth's response to COVID-19 challenges (Evans, 2020).

Furthermore, telerehabilitation fosters family involvement in the therapeutic process, especially for older adults and patients with limited mobility due to neurological conditions. This approach garnered high satisfaction rates and improved the overall quality of care during the pandemic (Chou et al., 2021). It also translates to time and cost savings for both patients and healthcare professionals by reducing transportation expenses and optimizing care delivery from the patient's home, enhancing accessibility for individuals with mobility limitations (Snoswell et al., 2020).

Patients recognized the importance of transitioning from in-person therapist-patient interactions to virtual settings to prevent COVID-19 transmission. This shift promoted consistent therapy, empowered patients to take charge of exercises at home, and supported treatment continuity, ultimately enhancing patient outcomes and progress (Serón et al., 2020; Villalobos Baeza et al., 2021).

### Future Directions

Although telerehabilitation is in its early stages in Colombia, it holds significant potential for the underserved population. This research underscores the importance of incorporating the perspectives of those involved in the practice, as understanding the needs of the people is crucial for effective healthcare delivery. Further research and the development of improved processes and policies are necessary to fully realize the benefits of telerehabilitation in the Colombian context.

### Strengths and Limitations

The limited literature on telerehabilitation in Colombia creates room for future development and has the potential to enhance overall healthcare. Drawing insights from examples in high-income countries, where telerehabilitation is more advanced, could offer valuable guidance in shaping the trajectory of telerehabilitation practices in Colombia.

As a qualitative study, there is no statistical or representative sample of the study population. Our data collection was based on the principle of information saturation. This qualitative research approach acknowledges the importance of subjectivity in obtaining in-depth and nuanced data. We primarily relied on verbal information to understand how patients and providers perceive the telerehabilitation services offered during the pandemic (López Estrada & Deslauriers, 2011).

Conversely, the absence of triangulation with additional data sources, such as document analysis, participant observation, or group interviews, may introduce potential inaccuracies in our conclusions. Nevertheless, it's crucial to highlight that our study encompassed a diverse population, incorporating viewpoints from both professionals and patients. Furthermore, a collaborative effort involving two researchers was integral to coding, analyzing, and discussing the results. This collaborative approach aimed to minimize biases in interpretations and to foster a more comprehensive understanding of telerehabilitation within the scope of this study.

## Conclusions

Telerehabilitation has proven to be a valuable instrument before, during, and after the pandemic, responding to the significant changes in healthcare systems both locally and globally. It has enhanced access to healthcare services, enabling outreach to the rural populations. This approach has extended the reach of care, benefiting individuals in remote areas or those with mobility limitations. Moreover, involving family members in the therapeutic process has demonstrated positive outcomes, fostering patient empowerment and overall care quality during these challenging times. A swift adaptation to virtual technologies has been necessary to optimize therapy sessions and yield time and cost savings for both patients and healthcare professionals by reducing transportation expenses.

In conclusion, telerehabilitation effectively improved the accessibility and timeliness of rehabilitation services during the pandemic. While there are certain limitations, such as a dearth of research on the perspectives of healthcare personnel and patients, this underscores the importance of further investigation, particularly in low- and middle-income countries. These studies can help address the challenges and ensure the delivery of quality care through virtual means, while acknowledging the ongoing need for refinement in this approach.

## References

[R1] Álvarez Castaño, L.S. (2009). Los determinantes sociales de la salud: más allá de los factores de riesgo. Revista Gerencia y Políticas de Salud, 8(17), 69–79. http://www.scielo.org.co/scielo.php?script=sci_abstract&pid=S1657-70272009000200005&lng=e&nrm=iso&tlng=es

[R2] Ambrosino, P., Papa, A., Maniscalco, M., Minno, M., & Nicola, D.D. (2021). COVID-19 and functional disability: current insights and rehabilitation strategies. Postgraduate Medical Journal, 97(1149), 469–470. 10.1136/postgradmedj-2020-138227 32753565 PMC10017007

[R3] Aryananda, R. A., Nieto-Calvache, A.J., Duvekot, J.J., Aditiawarman, A., & Rijken, M.J. (2023). Management of unexpected placenta accreta spectrum cases in resource-poor settings. American Journal of Obstetrics and Gynecology Global Reports, 3(2), 100191. 10.1016/j.xagr.2023.100191 37168547 PMC10165260

[R4] Avendaño Veloso, A., Parada Hernández, F., & Ortiz Contreras, J. (2022). Telemedicina para respuesta rápida a la pandemia COVID–19: Experiencia y lecciones aprendidas de una buena práctica para abordaje de crisis. Revista Internacional de Salud, Bienestar y Sociedad, 8(2), 55–63. 10.18848/2474-5219/CGP/v08i02/55-63

[R5] Begazo Flores, P., Supervía, M., Gimeno González, M., & Morata Crespo, A.B. (2023). Impacto de la pandemia por COVID-19 en los Servicios de Rehabilitación de España. Rehabilitación, 57(2), 100736. 10.1016/j.rh.2022.02.009 35545483 PMC8898680

[R6] Boldrini, P., Garcea, M., Brichetto, G., Reale, N., Tonolo, S., Falabella, V., Fedeli, F., Cnops, A. A., & Kiekens, C. (2020). Living with a disability during the pandemic. “Instant paper from the field” on rehabilitation answers to the COVID-19 emergency. European Journal of Physical and Rehabilitation Medicine, 56(3), 331–334. 10.23736/S1973-9087.20.06373-X 32406226

[R7] Boyle, C.A., Fox, M.H., Havercamp, S.M., & Zubler, J. (2020). The public health response to the COVID-19 pandemic for people with disabilities. Disability and Health Journal, 13(3), 100943. 10.1016/j.dhjo.2020.100943 32499132 PMC7246015

[R8] Camacho, Y.A. (2020). Cali realizó Foro virtual basado en la Telerehabilitación. https://www.cali.gov.co/salud/publicaciones/157181/cali-realizo-foro-virtual-basado-en-la-telerehabilitacion/

[R9] Castillo, V.S., Cano, C.A.G., & Gonzalez-Argote, J. (2023). Telemedicine and mHealth Applications for Health Monitoring in Rural Communities in Colombia: A Systematic Review. EAI Endorsed Transactions on Pervasive Health and Technology.

[R10] CEDLAS & The World Bank. (2021). Socio-Economic Database for Latin America and the Caribbean (CEDLAS and The World Bank). https://www.cedlas.econo.unlp.edu.ar/wp/en/estadisticas/sedlac/estadisticas/#1496165425791-920f2d43-f84a

[R11] Chang, M.D.L.P., Davancens, A., Rourich, M.C., Vincenti, J.M., Valencia, P., Guarriello, M.F., Costilla, C.M., Estol, C.J., Chang, M.D.L.P., Davancens, A., Rourich, M.C., Vincenti, J.M., Valencia, P., Guarriello, M.F., Costilla, C.M., & Estol, C J. (2021). Telemedicina en prevención secundaria y rehabilitación del accidente cerebrovascular durante la pandemia por COVID-19. Medicina (Buenos Aires), 81(3), 415–420. http://www.scielo.org.ar/scielo.php?script=sci_abstract&pid=S0025-76802021000300415&lng=es&nrm=iso&tlng=es 34137702

[R12] Chou, T.-J., Wu, Y.-R., Tsai, J.-S., Cheng, S.-Y., Yao, C.-A., Peng, J.-K., Chiu, T.-Y., & Huang, H.-L. (2021). Telehealth-based family conferences with implementation of shared decision making concepts and humanistic communication approach: A mixed-methods prospective cohort study. International Journal of Environmental Research and Public Health, 18(20), 10801. 10.3390/ijerph182010801 34682545 PMC8535301

[R13] ClinicalTrials.gov. Busqueda de Ensayos en el Registro: rehabilitation, telemedicine, COVID-19 Washington D.C. https://clinicaltrials.gov/ct2/results?cond=&term=rehabilitation%2C+telemedicine%2C+COVID-19&cntry=&state=&city=&dist=

[R14] Coronel, E., Candoni, G., Pelaez, S., Sanchez-Correa, C., Tomadín, R., & Valdez, M. (2022). Percepciones sobre la rehabilitación durante la pandemia por COVID-19 de las personas con discapacidad motora. Rehabilitación, 100737. 10.1016/j.rh.2022.03.002 PMC9035362 36357221

[R15] DANE. (2023). Comunicado de prensa Encuesta Nacional de Calidad de Vida (ECV) 2022

[R16] de Sire, A., Andrenelli, E., Negrini, F., Negrini, S., & Ceravolo, M.G. (2020). Systematic rapid living review on rehabilitation needs due to COVID-19: update as of April 30th, 2020. European Journal of Physical and Rehabilitation Medicine, 56(3), 354–360. 10.23736/S1973-9087.20.06378-9 32408729

[R17] de Varge Maldonado, J.M.S., de Paula, A.C., & Gadelha, C.A.G. (2023). Perception of health care providers and users on teleconsultation in times of COVID-19 in Brazil: An exploratory interview study. Telemedicine journal and e-health, 29(5), 717–725. 10.1089/tmj.2022.0132 36282808

[R18] Eraso, J.A.F. (2015). Diagnóstico y rehabilitación neuropsicológica de los traumatismos craneoencefálicos. Una necesidad por atender en Colombia. Tesis Psicológica, 10(2), 86–103. https://revistas.libertadores.edu.co/index.php/TesisPsicologica/article/view/631

[R19] Escobar, M. F., Gallego, J.C., Echavarria, M.P., Fernandez, P., Posada, L., Salazar, S., Gutierrez, I., & Alarcon, J. (2023). Maternal and perinatal outcomes in mixed antenatal care modality implementing telemedicine in the southwestern region of Colombia during the COVID-19 pandemic. BMC Health Services Research, 23(1), 259. 10.1186/s12913-023-09255-4 36922841 PMC10017345

[R20] Evans, A. (2020). Rapid learning curve with telehealth; a clinical audit at the time of 'flattening the infection curve' during the coronavirus (SARS Cov-2) pandemic. The Foot and Ankle Online Journal, 13, 7. 10.3827/faoj.2020.1303.0007

[R21] Figueroa, L.M. (2020). Telehealth in Colombia, challenges associated with COVID-19. Biomédica, 40, 77–79. http://www.scielo.org.co/scielo.php?script=sci_arttext&pid=S0120-41572020000600077&nrm=iso 33152191 10.7705/biomedica.5594 PMC7676832

[R22] Fundacion IDEA. (2022). Informe de Gestion 2022. https://www.fundacionideal.org.co/sites/default/files/imce/informe_de_gestion_presidencia_ejecutiva_2022.pdf

[R23] Gallegos S.R.L. (2020). Telesalud y telemedicina para la prestación de servicios de salud en la pandemia por Covid-19 GESTION DE PRESTACIÓN DE SERVICIOS EN SALUD.

[R24] García-Pereáñez, J., & García-Arango, D. (2021). Legal ethical implications in the exercise of communication and information technologies (ICT) in telemedicine and e-law in Medellín - Colombia. Trends and Applications in Information Systems and Technologies.

[R25] Gillin, J. (2015). Understanding the causes of Colombia's conflict: Inequality. Colombia Reports. https://colombiareports.com/understanding-colombias-conflict-inequality/

[R26] Gobierno de Colombia. (2019). Ley No. 1419, 13 de Diciembre de 2010, “Por La Cual Se Establecen Los Lineamientos Para El Desarrollo De La Telesalud En Colombia”. 2019.

[R27] Gómez Acosta, C. A., & Cuervo Echeverri, C. (2007). Conceptualización de discapacidad: reflexiones para Colombia. Universidad Nacional de Colombia. Facultad de Medicina. https://repositorio.unal.edu.co/handle/unal/70325

[R28] Guest, G., Bunce, A., & Johnson, L. (2006). How many interviews are enough? An experiment with data saturation and variability. Field Methods, 18(1), 59–82. 10.1177/1525822X05279903

[R29] Haleem, A., Javaid, M., Singh, R.P., & Suman, R. (2021). Telemedicine for healthcare: Capabilities, features, barriers, and applications. International Journal of Remote Sensing, 2, 100117. 10.1016/j.sintl.2021.100117 PMC8590973 34806053

[R30] Hernández-Zambrano, S., Castiblanco-Montañez, R., Chávez, J., Rivera-Triana, D., Aza, A., Villarreal, L., Martinez, M., Rojas-Villarraga, A., & Santos-Moreno, P. (2021). Unexpected difference in acceptance of teleconsultation between patients with lupus and rheumatoid arthritis who underwent to a developed and implemented telemedicine innovative program after the Declaration of Quarantine due to the Covid-19 pandemic in Colombia. Annals of the Rheumatic Diseases, 80, 1475.1473–1476. 10.1136/annrheumdis-2021-eular.389934215646

[R31] Jalali, M., Shahabi, S., Bagheri Lankarani, K., Kamali, M., & Mojgani, P. (2020). COVID-19 and disabled people: perspectives from Iran. Disability & Society, 35(5), 844–847. 10.1080/09687599.2020.1754165

[R32] Leocani, L., Diserens, K., Moccia, M., Caltagirone, C., & Neurorehabilitation Scientific Panel of the European Academy of Neurology, E. A. N. (2020). Disability through COVID-19 pandemic: neurorehabilitation cannot wait. European Journal of Neurology, 27(9), e50–e51. 10.1111/ene.14320 32402100 PMC7273105

[R33] López Estrada, R.E., & Deslauriers, J.-P. (2011). La entrevista cualitativa como técnica para la investigación en Trabajo Social. Margen: revista de trabajo social y ciencias sociales(61), 2–19. https://dialnet.unirioja.es/servlet/articulo?codigo=3756178

[R34] Marín Urrego, J.C.M.L., Socorro; Peña Torres, Esperanza; Mariño, Javier; Martínez-Álvarez, Eddier; Duque Yara, Nidia & Cadena-Camargo, Yazmín (2023). Defining differential approach and intersectional perspective: A multimethod study. Universitas Medica, 64(1). https://revistas.javeriana.edu.co/files-articulos/UMED/64-1(2023)/231073960015/index.html

[R35] Marks, D., Kitcher, S., Attrazic, E., Hing, W., & Cottrell, M. (2022). The Health Economic Impact of musculoskeletal physiotherapy delivered by telehealth: A systematic review. International Journal of Telerehabilitation, 14(2). 10.5195/ijt.2022.6524 PMC10681044 38026565

[R36] Michell, A., Besomi, M., Seron, P., Voigt, M., Cubillos, R., Parada-Hernández, F., Urrejola, O., Ferreira-Pacheco, T.B., De Oliveira-Silva, D., Bianca Aily, J., Moreno-Collazos, J. E., Pinzón-Ríos, I.D., Aguirre-Aguirre, C.L., Hinman, R.S., Bennell, K.L., & Russell, T. G. (2022). Implementation of physiotherapy telerehabilitation before and post Covid-19 outbreak: A comparative narrative between South American countries and Australia. Salud Publica Mex, 64, S31-s39. 10.21149/13160 36130385

[R37] Mora-Moreo, L., Estrada-Orozco, K., Espinosa, O., & Melgarejo, L. M. (2023). Characterization of the population affiliated to the subsidized health insurance scheme in Colombia: a systematic review and meta-analysis. International Journal for Equity in Health, 22(1), 28. 10.1186/s12939-022-01818-x 36747197 PMC9903445

[R38] Moreno-Chaparro, J., Calderón-Calvo, A., Cubillos-Mesa, C., & Moreno-Angarita, M. (2018). Política y práctica: servicios de fonoaudiología y terapia ocupacional para la primera infancia colombiana. Revista de la Facultad de Medicina, 66, 97–102. http://www.scielo.org.co/scielo.php?script=sci_arttext&pid=S0120-00112018000100097&nrm=iso

[R39] Narváez, F., Marín-Castrillón, D.M., Cuenca, M.C., & Latta, M.A. (2017). Development and implementation of technologies for physical telerehabilitation in Latin America: A systematic review of literature, programs and projects. TecnoLógicas, 20, 155–176. http://www.scielo.org.co/scielo.php?script=sci_arttext&pid=S0123-77992017000300012&nrm=iso

[R40] Organisation for Economic Co-operation and Development. (2022). OECD Rural Policy Review of Colombia. Secretary-General of the OECD. https://www.oecd.org/regional/rural-development/Rural-Policy-Review-Colombia-PH-EN.pdf

[R41] Ospina, J., & Serrano, F. (2010). El paciente amputado: complicaciones en su proceso de rehabilitación. Revista Ciencias De La Salud, 7(2). 10.12804/revistas.urosario.edu.co/revsalud/a.276

[R42] Pereáñez, J.A.G., Arango, D.A.G., Villa, C.F.H., Aguirre, J.A.S., Giraldo, L.F.G., & Cardona, M.A.M. (2019). Situations about telemedicine in Colombia: Between the legal and the legitimate. 2019 14th Iberian Conference on Information Systems and Technologies (CISTI),

[R43] Qvarfordt, M., Nilsson, E., & Nilsson, L. (2023). Health care professionals' experiences in telerehabilitation: Qualitative content analysis. Journal of Medical Internet Research Human Factors, 10, e40690. 10.2196/40690 PMC10157457 37074772

[R44] Rubiano-Ovalle, O., García-Melo, J., & Claro-Candezano, M. (2022). Atención sincronizada de tele rehabilitación en zonas rurales con apoyo de ayudas tecnológicas tele operadas: aplicación a un caso Colombiano. Investigación e Innovación en Ingenierias, 10. 10.17081/invinno.10.2.5652

[R45] Savedoff, W., Goyeneche, L., Soler, L., Bernal-Mariángela, P., Chávez, J., & Cardona, L. (2023). Disruption and rebound: Healthcare and telemedicine in Colombia during the COVID-19 pandemic for chronic care patients Inter-American Development Bank.. 10.18235/0004865

[R46] Scott, R.E., & Mars, M. (2020). Response to Smith et al.: Telehealth for global emergencies: Implications for coronavirus disease 2019 (COVID-19). Journal of Telemedicine and Telecare, 26(6), 378–380. 10.1177/1357633X20932416 32525438

[R47] Serón, P., Oliveros, M.-J., Fuentes-Aspe, R., & Gutiérrez-Arias, R. (2020). Effectiveness of telerehabilitation in physical therapy: A protocol for an overview in a time when rapid responses are needed. Medwave, 20(7), e7970. 10.5867/medwave.2020.07.7970 32804923

[R48] Silva-Tinoco Rubén, T.-S.V. d. l. (2021). La imperiosa necesidad de telemedicina en la atención de diabetes durante la pandemia de COVID-19. Un estudio de abordaje integral. Gaceta médica de México, 157. https://www.scielo.org.mx/scielo.php?script=sci_arttext&pid=S0016-38132021000300323 10.24875/GMM.M21000563 34667324

[R49] Snoswell, C.L., Taylor, M.L., Comans, T.A., Smith, A.C., Gray, L.C., & Caffery, L.J. (2020,. Determining if telehealth can reduce health system costs: Scoping review. Journal of Medical Internet Research, 22(10), e17298. 10.2196/17298 33074157 PMC7605980

[R50] Sugarman, D.E., Busch, A.B., McHugh, R K., Bogunovic, O.J., Trinh, C.D., Weiss, R.D., & Greenfield, S. F. (2021) Patients' perceptions of telehealth services for outpatient treatment of substance use disorders during the COVID-19 pandemic. The American Journal on Addictions, 30(5), 445–452. 10.1111/ajad.13207 34405475 PMC8429128

[R51] Trejos-Herrera, A.M., Vinaccia, S., & Bahamón, M.J. (2020). Coronavirus in Colombia: Stigma and quarantine. Journal of Global Health, 10(2), 020372. 10.7189/jogh.10.020372 33110564 PMC7568913

[R52] Villalobos Baeza, E., Alonso Álvarez, B., & Palomino Aguado, B. (2021). El servicio de rehabilitación en la pandemia por COVId-19: adaptaciones y nuevos retos. Rehabilitación, 55(2), 86–88. 10.1016/j.rh.2020.10.007 33632472 PMC7609042

[R53] World Health Organization, W. (2020). Nuevo coronavirus 2019. https://www.who.int/es/emergencies/diseases/novel-coronavirus-2019; https://www.who.int/es/emergencies/diseases/novel-coronavirus-2019?gclid=Cj0KCQjw7ZL6BRCmARIsAH6XFDKcI6LsxBCxMw7iuSp1hu-a_NzTP1QoftRyg_xnnC07x8tHnNk1L_MaAmm7EALw_wcB

